# Preclinical Imaging for the Translation of Innovative Nutritional Strategies: Current Applications and Future Perspectives

**DOI:** 10.3390/jimaging12090444

**Published:** 2026-09-15

**Authors:** Ippei Yamaoka

**Affiliations:** Medical Foods Research Institute, Otsuka Pharmaceutical Factory, Inc., Muya-cho, Naruto 772-8601, Japan; yamaokai@otsuka.jp; Tel.: +81-88-684-2228; Fax: +81-88-686-8185

**Keywords:** fluorescence imaging, residual lipid emulsion, catheter flushing, feeding tube, microbial proliferation, gastrointestinal transit, pectin, nutrition retention, optical mapping, clinical nutrition systems

## Abstract

Nutrient persistence, which manifests as residual deposits within parenteral and enteral delivery systems and as prolonged intraluminal retention in the gastrointestinal (GI) tract, represents a central determinant of safety and performance in clinical nutrition. This review integrates evidence across parenteral nutrition (PN), enteral nutrition (EN), and GI physiology to provide a comprehensive and mechanistic analysis of how nutrient residues arise and may affect clinically relevant outcomes among diverse nutrition delivery settings. Particular emphasis is placed on the role of imaging-based preclinical research in linking mechanistic observations of nutrient persistence with translational questions relevant to clinical nutrition. Traditional assessments of nutrient persistence have relied largely on indirect or bulk endpoints, limiting the ability to determine where nutrients remain, how long they persist, and how effectively they are cleared. Recent advances in fluorescence-based imaging provide spatially resolved and longitudinal approaches for assessing nutrient behavior in benchtop systems and preclinical models, revealing how transport dynamics and device geometry affect residue accumulation in PN systems, formulation rheology and thickener chemistry affect residue formation and microbial proliferation in EN delivery pathways, and physicochemical transformations and motility govern GI retention. This review synthesizes findings across these domains to establish an imaging-guided framework that links device engineering, formulation science, and physiological processes. Importantly, fluorescence-based imaging is presented in this review as a complementary approach rather than a replacement for established methods. Its translational value lies in its potential to generate measurable indicators of residue formation, clearance, and retention that may be linked to outcomes such as catheter performance, microbial proliferation, feeding tolerance, and nutrition delivery efficiency. Fluorescence-imaging supports the 3Rs (Replacement, Reduction, and Refinement) by enabling optimization in benchtop PN/EN models before animal studies and by allowing longitudinal assessment in GI animal models. Although challenges related to probe performance, standardization, and clinical validation remain, this approach positions nutrient persistence as a measurable and potentially modifiable parameter. Future research should prioritize standardized, outcome-linked preclinical imaging approaches that support clinical translation while promoting more efficient and responsible experimental design.

## 1. Introduction

A single cross-cutting challenge persists across modern clinical nutrition: the continued presence of nutrient residues within parenteral nutrition (PN) devices, enteral nutrition (EN) systems, and the gastrointestinal (GI) tract. Whether occurring as microscopic deposits on the internal surfaces of parenteral and enteral devices or as prolonged intraluminal retention within the GI tract, these manifestations of nutrient persistence may compromise clinical outcomes through device dysfunction, microbial proliferation, feeding intolerance, and secondary infections [1,2,3]. In GI physiology, delayed gastric emptying, impaired propulsion, and prolonged exposure of nutrients to intestinal environments have long been recognized as contributors to intolerance, dysmotility, and symptom generation [4]. Although these phenomena occur in distinct clinical settings, nutrient persistence in PN devices, EN systems, and the GI tract is governed by shared principles of transport, clearance, and physicochemical interactions.

Published guidelines spanning documents across parenteral, enteral, and home-nutrition practice emphasize the importance of trained operators, standardized maintenance procedures, and programmatic oversight [5,6,7]. However, despite these recommendations, a persistent methodological gap has remained. Although routine operational variables, such as flushing technique, flow sequence, flow rate, and infusion-set geometry, are widely acknowledged to affect device performance, earlier methodologies were unable to directly determine whether nutrients were effectively cleared or persisted within artificial delivery systems [8,9]. Accordingly, many long-standing recommendations relied on accumulated clinical experience rather than direct observational evidence, a limitation that is clinically significant. Minute nutrient residues undetectable by visual inspection can create biophysically stable microenvironments that promote biofilm formation, alter fluid dynamics and downstream nutrient exposure [1,2,3]. Comparable problems arise within the GI tract, where delayed emptying and prolonged nutrient residence affect microbial activity, segmental transit, and overall tolerance, as emphasized in comprehensive reviews of GI motor physiology and noninvasive motility assessment [4]. In both artificial and biological systems, nutrient persistence has historically been inferred from downstream consequences rather than direct observation, because methods capable of visualizing nutrient accumulation and residence over time were not available.

Despite substantial progress within each domain of clinical nutrition, the literature has largely remained confined to individual segments of the nutrition pathway. Reviews of PN have primarily focused on catheter-related complications and infection prevention strategies [10,11], whereas EN studies have emphasized contamination and feeding-system management [12,13]. In contrast, GI research has centered on GI motility and transit physiology [14,15]. Although these contributions have advanced their respective fields, they rarely address nutrient persistence as a shared mechanistic process spanning artificial devices and biological systems.

The key strength of this review lies in its integration of PN devices, EN delivery systems, and GI physiology under the unified concept of nutrient persistence. By applying an imaging-guided perspective, this review connects device-level, system-level, and GI phenomena and highlights nutrient persistence as a shared process across artificial and biological systems. A central aim is to evaluate how preclinical imaging can facilitate the translation of nutrient persistence research into clinically relevant strategies for optimizing nutrition delivery and improving patient outcomes. Recent advances in imaging-based methodologies, particularly fluorescence-based approaches, now enable direct visualization of residual nutrient material in catheters and feeding tubes and the tracking of nutrient residence within the GI tract. These technologies provide spatially resolved and quantitative assessment of nutrient persistence across the continuum of clinical nutrition, offering mechanistic insights that are difficult to obtain using conventional methodologies [16,17,18,19].

Beyond their mechanistic and clinical value, these imaging approaches also offer practical advantages for preclinical nutrition research through their alignment with the principles of the 3Rs (Replacement, Reduction, and Refinement) [20,21]. By enabling optimization in benchtop systems and non-invasive longitudinal assessment in animal models, imaging-based approaches, particularly fluorescence imaging, can reduce animal use, minimize invasive procedures, and improve experimental efficiency. These features also enable repeated measurements within the same animal, reducing inter-animal variability while improving statistical robustness and data quality. Collectively, such capabilities position imaging-based methodologies as translational technologies that advance both nutritional science and responsible animal research.

This review was conducted as a narrative review. Literature searches were performed using PubMed and Google Scholar, and the final selection was guided by the conceptual structure of the review. Publications were identified using combinations of terms related to PN, EN, feeding tubes, catheters, nutrient persistence, nutrient residues, retention, GI transit, gastric emptying, and imaging-based assessment, including fluorescence imaging, MRI, scintigraphy, and breath tests. The literature was then organized to examine nutrient persistence sequentially across PN devices, EN delivery systems, and GI physiology, with particular attention to studies that provided mechanistic links between these traditionally separate fields. Additional references were identified through citation tracking. Studies were included when they offered methodological, mechanistic, clinical, or translational insight into nutrient persistence and its assessment. It should be noted that this review was not conducted as a systematic review, and no formal risk-of-bias assessment or meta-analysis was performed.

## 2. Device-Level Persistence: Parenteral Nutrition as a Model of Transport-Driven Residue Formation

The evidence reviewed in this section originates primarily from benchtop fluidic models of clinical PN administration and focuses on nutrient transport, residue formation, and catheter clearance under controlled experimental conditions. Historically, PN-related research has focused primarily on catheter-related complications rather than direct assessment of intraluminal nutrient residues. Accordingly, the mechanisms by which nutrient formulations adhere to internal surfaces and resist clearance have remained incompletely characterized. This section examines how imaging-based approaches enable direct quantification and mechanistic understanding of residue persistence in PN systems.

Before the introduction of imaging-based approaches, investigators relied on microbiological culture, scanning electron microscopy, turbidity measurements, and chemical analyses of flush effluent to assess PN-related deposition. While these methods have provided important insights into microbial risk and formulation stability, they offer limited information regarding the spatial distribution and persistence of nutrient residues within functional catheters.

Parenteral nutrition catheters provide well-characterized examples of how nutrient residues form within artificial delivery systems. Studies in clinical microbiology and infection control have demonstrated that PN solutions, including intravenous lipid emulsions, can support microbial growth and are associated with catheter-related bloodstream infection risk [1]. Mechanistic investigations further show that skin flora, hub contamination, and biofilm-forming organisms, including Staphylococcus spp. and Candida spp., colonize catheter surfaces through extracellular polymeric substance (EPS) production and biofilm development [2]. In parallel, device-level analyses have reported intraluminal colonization and biofilm formation on catheter surfaces, suggesting that conventional microbiological approaches may underestimate the true microbial burden [1,10].

Overall, these findings reinforce concerns that nutrient-rich infusates and the resulting residual deposits can sustain microbial communities within catheter systems. Subsequent attempts to evaluate PN-related deposition more directly encountered methodologic limitations. Microscopy of PN formulations and tubing segments revealed lipid globules or precipitates, but did not provide lumen-specific localization and could not determine whether residues persisted after flushing. Optical microscopy studies of PN stability quantified droplet enlargement and particulate formation under ex vivo conditions instead of within functional catheters. Turbidimetric and chemical analyses of discarded flush effluent provided only bulk measurements of lipid content without identifying the specific regions where residues remained. Molecular assays such as PCR detection of microbial DNA in catheter lumens quantified biological remnants rather than nutrient residues and could not address the underlying mechanistic questions. Moreover, comparisons of culture-based techniques with SEM in PN catheters have highlighted substantial false-negative rates for intraluminal colonization, underscoring the need for spatially resolved methods inside actual devices [1,2,8,9]. Consequently, although catheter-related infection mechanisms have been extensively investigated, the spatial behavior and persistence of nutrient residues within functioning catheter systems remained poorly understood. Contemporary reviews of catheter-related infections further emphasize that conventional antimicrobial or antiseptic strategies often provide only short-term suppression of biofilm, rather than addressing the upstream conditions that allow residues and EPS-mediated communities to establish and persist [2].

These methodological limitations highlight the need for techniques capable of directly visualizing intraluminal residue distribution in intact devices. Fluorescence imaging has emerged as a powerful approach for visualizing spatial heterogeneity in complex biological systems and therefore offers a unique opportunity to address this gap [22]. Using indocyanine green-labeled lipid emulsions, fluorescence imaging studies demonstrated that flushing practices and device configuration significantly affect residual lipid accumulation and clearance efficiency within clinically relevant catheter systems [16]. Fluorescence-based analyses showed that residual lipid deposits can persist despite routine flushing and accumulate preferentially in specific regions determined by flow dynamics and device geometry. These findings provide direct evidence that nutrient persistence is affected by transport conditions and device design and support the need for evidence-based catheter maintenance strategies [1,2,10].

Collectively, these findings position PN catheters as a tractable model for studying transport-driven residue formation in nutrient delivery systems. Rather than being determined solely by nutrient composition, residue persistence emerges from complex interactions among flow dynamics, flushing conditions, and device geometry. Imaging-based approaches make these otherwise hidden processes directly observable, revealing spatially heterogeneous patterns of nutrient retention within functional catheter systems. By enabling direct visualization of where residues accumulate and persist, imaging provides mechanistic insight into the transport processes underlying residue formation and clearance. This capability advances understanding beyond contamination or occlusion and supports a more mechanism-oriented approach to catheter maintenance, device design, and complication prevention.

## 3. System-Level Persistence: Enteral Feeding Tubes as Residue-Prone Biological Environments

The evidence summarized in this section includes predominantly benchtop and ex vivo investigations of feeding tubes, supplemented by selected clinical observations related to contamination, residue formation, and tube performance. Enteral nutrition feeding systems represent a distinct but closely related model of system-level nutrient persistence, as feeding tubes are repeatedly exposed to nutrient-dense formulations and reused over prolonged periods. Previous reviews have largely focused on contamination, handling practices, infection risk, and device-associated biofilms [12,13,23]. In contrast, the internal lumen of feeding tubes has often remained an analytical “black box,” with limited understanding of where nutrients persist after routine flushing or how small residual deposits contribute to microbial amplification. This section therefore examines how formulation properties, tube characteristics, and microbial contamination collectively affect residue persistence within EN delivery pathways.

Population-level evidence indicates that microbial contamination of EN products is common. A recent systematic review of 1099 preparations found that 44.7% exceeded acceptable bacterial thresholds overall, with the highest rates observed in home-prepared feeds; handling practices and storage conditions explained much of the risk [13]. Complementing these findings, a systematic review of blenderized tube feeding reported generally higher contamination rates than commercially prepared formulas and identified preparation practices, storage duration, and handling conditions as major determinants of microbial burden [23]. These studies suggest that contamination risk arises not only from the nutritional substrate itself but also from the conditions under which enteral formulations are prepared, handled, and administered.

Beyond large-scale contamination surveys, prospective clinical investigations in pediatric and neonatal settings have also identified the enteral tube hub and administration sets as critical reservoirs that seed downstream colonization. In a pediatric institution, administration sets frequently harbored the same microbes recovered from the hub [24], while molecular epidemiology demonstrated genetic relatedness between isolates from hubs and distal tubing, establishing a hub-to-set transmission route in clinical practice [25]. Neonatal observations further suggest that feeding tubes can contain high loads of potentially pathogenic and antibiotic-resistant bacteria, with dwell time and route of insertion shaping the biofilm community. Taken together, these findings clarify how microorganisms spread through hardware and patient interfaces, but they do not, by themselves, identify or quantify the nutrient residues that sustain such colonization after routine flushing.

Unlike research on PN systems, research on EN systems has rarely examined the spatial distribution of nutrient residues within feeding tube lumens. Prior studies have largely focused on formula characteristics, gastric residual volume, contamination of enteral feeding solutions, and device-related complications [12,13,26,27], leaving the spatial distribution of intraluminal nutrient residues poorly characterized. Imaging-based analyses using fluorescence and bioluminescence revealed that nutrient residues persist at multiple intraluminal sites even after routine flushing and that microbial growth closely follows the spatial distribution of these residual substrates [17]. The marked heterogeneity observed along the tube length highlights a key limitation of conventional approaches, which lack the lumen-wide spatial resolution required to quantify residue persistence in EN systems. Taken together, these findings highlight the need for analytical methods capable of mapping nutrient residue distribution throughout the entire feeding tube, complementing conventional bulk measurements.

Four mechanistic observations from the current research on EN emerge. First, residue burden increases with formulation viscosity and thickener chemistry, indicating a direct rheology–residue relationship. Second, microbial bioluminescence consistently co-localizes with fluorescent residues, showing that even small amounts of remaining substrate are biologically significant. Third, the intrinsic fluorescence of many EN formulations enables quantitative tracking of residue clearance without modification of the feed matrix, facilitating direct assessment of persistence dynamics. Fourth, higher-viscosity formulations require substantially more flushing to achieve complete clearance, explaining why tubing that appears visually clean may still contain detectable nutrient residues [17].

Considered together, these findings shift EN tube management from a visually guided routine to a quantifiable, formulation-aware process. Broad surveillance studies define the magnitude and upstream determinants of risk [12,13]. Device-focused investigations of hubs and administration sets delineate the routes by which organisms spread and how dwell time and tube route modulate biofilm communities [26,27]. At the mechanistic level, fluorescence-based tube mapping provides the missing spatial link between formulation chemistry, residue persistence, and microbial amplification. These insights support formulation-specific, viscosity-informed flushing strategies and evidence-based tube care protocols.

More broadly, these observations suggest that residue persistence in EN systems is fundamentally a transport-dependent phenomenon arising from interactions among formulation properties, flow conditions, delivery practices, and device architecture. Nutrient retention and clearance are therefore shaped not only by the composition of the formula itself but also by the transport environment through which it is delivered. By providing spatially resolved assessment of these processes, imaging studies reveal otherwise hidden patterns of nutrient accumulation and clearance and offer a mechanistic framework for understanding nutrient behavior within feeding systems. Such insights support more rational approaches to EN delivery, device optimization, and prevention of nutrition-related complications.

## 4. GI-Level Persistence: Imaging Nutrient Transit and Retention in the Gastrointestinal Tract

In contrast to the PN and EN sections, the studies reviewed for this section were performed primarily in animal models and focused on in vivo assessment of GI retention, transit, and motility. Understanding how long nutrients reside within the GI tract and how their movement is spatially regulated is central to both physiological research and clinical management of feeding tolerance. This section critically evaluates current methodologies for assessing GI transit and retention and highlights fluorescence-based imaging as a unifying framework for spatially resolved evaluation of GI nutrient persistence.

Conventional GI motility assessments, including scintigraphy, magnetic resonance imaging, marker-based assays, and 13C breath tests, have contributed substantially to the field by enabling quantitative and translational evaluation of transit kinetics. Each method has strengths for assessing specific aspects of GI function; however, they often provide only partial information on the spatial distribution and retention of nutrients under dynamic physiological conditions. Consequently, no single conventional approach can comprehensively characterize where nutrients remain and how their persistence changes over time within the GI tract.

Fluorescence-based GI imaging offers a complementary perspective by enabling noninvasive, spatially resolved visualization of nutrient behavior across multiple physiological timescales. Both fluorescence- and bioluminescence-based imaging approaches have been validated as noninvasive methods for assessing gastric emptying and GI transit in small-animal models. For example, a bioluminescence-based gastric-emptying mouse model enabled repetitive in vivo assessment of gastric emptying without the need for terminal procedures, demonstrating the potential of optical imaging for longitudinal GI functional studies [28]. Recent advances in miniaturized fluorescence imaging technologies further support direct intraluminal assessment of GI processes and may expand future opportunities for high-resolution evaluation of nutrient persistence [29]. By directly tracking labeled or intrinsically fluorescent formulations, these approaches reveal where nutrients remain, how long they persist, and how physicochemical transformations, intestinal propulsion, and formulation properties affect their retention and clearance within the GI tract.

Among conventional approaches, rapid magnetic resonance imaging (MRI) enables noninvasive evaluation of gastric emptying and coordinated gastroduodenal motility in humans. Teramoto et al. demonstrated that rapid MRI can quantify changes in gastric fundal area, duodenal wall motion velocity, and gastric-duodenal coordination following ingestion of a liquid meal, providing simultaneous assessment of gastric emptying and upper GI motor function [30]. Although MRI has been applied in both human and rodent studies, its high cost and operational complexity limit its practicality for high-throughput formulation research [31].

Scintigraphy remains a clinical standard for assessing gastric emptying and whole-gut transit because radiolabeled meals enable quantitative, geometry-independent measurement of GI transit. Pinhole gamma camera techniques have extended this approach to murine models, demonstrating strong correlation with phenol red assays while permitting serial within-subject measurements [32]. However, the requirement for radiotracers, dedicated instrumentation, and specialized facilities limits widespread adoption.

Marker-based assays (e.g., carmine red) are widely used because of their simplicity and accessibility, but they provide only discrete time-point measurements and therefore limited regional resolution. Methodological refinements, including dark-phase testing, home- or pair-housing, UV-fluorescent markers, combined carmine/FITC-dextran approaches, and community-developed SOPs, have improved reproducibility and reduced handling-related variability [33,34]. Nevertheless, these methods remain indirect and cannot visualize intraluminal nutrient distribution in situ.

^13^C breath tests enable repeatable, noninvasive assessment of gastric emptying in rodents and have clear translational relevance. However, their application is limited by the need for specialized breath-collection systems, relatively low throughput, and costly analytical platforms such as infrared spectrometry or gas chromatography–mass spectrometry [35]. In addition, technical constraints have historically restricted longitudinal assessment of gastric emptying in disease models, limiting their utility for monitoring progression over extended periods.

Fluorescence-based GI imaging fills this methodological gap by providing spatially and temporally resolved assessment of nutrient persistence across multiple physiological timescales. Gastric emptying (minutes to hours) can be visualized following administration of a near-infrared-labeled liquid meal; in mice, pectin-containing EN forms a gel in the acidic gastric environment and empties more slowly than pectin-free EN [18]. Whole-gut transit (several hours) can be quantified using fecal indocyanine green imaging, which defines transit by the appearance of the first fluorescent feces, detects dose-dependent atropine-induced delays, and correlates strongly with carmine red measurements. Under liquid-diet conditions, low-methoxyl pectin normalizes transit, whereas high-methoxyl pectin prolongs it [19]. Together, these findings demonstrate that fluorescence-based approaches can quantify nutrient persistence across multiple stages of GI transport, from gastric emptying to whole-gut transit.

These optical approaches align with ongoing efforts to establish noninvasive and translational motility methods that better reflect clinical physiology. By showing where and for how long nutrients remain within the GI tract, they provide mechanistic insight directly relevant to clinical nutrition. This improves the interpretability of preclinical findings and supports the design of formulation and delivery strategies that enhance feeding tolerance. Nevertheless, several technical challenges remain before these methods can be applied more broadly across diverse experimental settings. Extending imaging to longer observation windows will require further probe optimization, including the development of chemically modified indocyanine green (ICG) derivatives that maintain signal stability during prolonged monitoring [36,37,38,39,40,41,42,43].

Beyond its utility for assessing GI transit, fluorescence-based imaging offers important advantages from a 3Rs perspective. In contrast to conventional endpoint-based approaches, such as dye-marker assays, which often require separate animal cohorts at multiple time points, fluorescence imaging enables longitudinal assessment within the same animal. This capability increases data yield per animal, reduces inter-animal variability, decreases overall animal use, and minimizes reliance on invasive or terminal procedures, thereby directly supporting the principles of Reduction and Refinement [20,21].

Collectively, these studies highlight the GI tract as a dynamic transport environment in which nutrient fate is shaped by the interaction of formulation properties, physiological function, and local flow behavior. Imaging makes these processes directly observable, enabling spatially and temporally resolved assessment of nutrient transit, distribution, and clearance. This mechanistic insight goes beyond traditional evaluations of GI function and provides a basis for imaging-guided approaches to nutritional assessment, intervention, and precision nutrition.

To place these advantages in context, Table 1 summarizes representative imaging and non-imaging approaches using common comparison criteria, including principle, strengths, limitations, translational relevance, and relevance to the 3Rs. This format is intended to highlight the complementary roles of different methodologies rather than to rank one approach above another.

## 5. Discussion

The evidence synthesized across PN, EN, and GI physiology suggests that nutrient persistence represents a common process linking device performance, nutrient delivery, microbial proliferation, and GI tolerance. Although these systems have traditionally been investigated separately, the studies examined in this narrative review indicate that residue formation in artificial nutrition devices and prolonged nutrient retention within the GI tract can be interpreted within a shared framework of transport, clearance, and physicochemical interaction. This perspective also highlights the limitations of conventional endpoint-based assessments. Direct visualization now makes it possible to examine where nutrients accumulate, how long they persist, and how these processes relate to clinically relevant outcomes.

Fluorescence-based imaging provides a complementary perspective by enabling direct, spatially resolved visualization of nutrient behavior. Recent reviews have highlighted both the strengths and current technical limitations of fluorescence imaging [40,41,42,43]. Across PN, EN, and GI systems, imaging permits direct observation of processes that were previously inferred only indirectly, including residue formation, clearance efficiency, and prolonged nutrient retention. Together, these observations demonstrate that nutrient persistence can be measured across diverse nutritional settings using a common spatially resolved framework.

Importantly, imaging-derived measures of nutrient persistence may help bridge mechanistic observations and clinically relevant outcomes. In PN systems, persistent residues can be considered in relation to catheter performance, occlusion risk, biofilm formation, and catheter-related infections, because biofilms and microorganisms have been reported on central venous catheters used for PN and may indicate a predisposition to infection [1]. In EN delivery pathways, residue accumulation may provide substrates for microbial proliferation while also influencing nutrient delivery efficiency and tube clearance; this interpretation is consistent with systematic reviews showing that microbiological contamination of enteral tube feeding remains a relevant safety issue in hospital and home settings [12,13]. In the GI tract, imaging-derived assessments of gastric emptying and GI transit may provide mechanistic insight into delayed nutrient movement, altered transit kinetics, and feeding intolerance [44,45]. Although further validation is required, these imaging-derived metrics may serve as translational indicators for evaluating interventions aimed at improving safety and performance in clinical nutrition.

At the same time, fluorescence-based imaging is not without limitations and should therefore be interpreted as a complementary approach rather than a replacement for established methods. Although it enables spatially resolved and longitudinal assessment of nutrient behavior, its application is limited by tissue penetration, probe stability, signal attenuation, quantitative interpretation, and lack of standardized protocols [36,37,38,39,40,41,42,43]. Generalizability across different devices, formulations, and species also remains limited, as most studies have been conducted under controlled experimental conditions. Accordingly, the choice of method should depend on the specific research question, biological system, and clinical context [40,41,43].

The evidence discussed in this review includes both established findings and emerging imaging-based observations. Established evidence from clinical studies, systematic reviews, and practice guidelines supports the importance of addressing catheter-related complications, enteral feeding tube contamination, microbial proliferation, and GI transit disorders. By contrast, fluorescence-based assessment of nutrient persistence provides direct evidence regarding residue formation, clearance, and retention but remains an emerging methodology that requires further independent validation. Several examples discussed in this review originate from the author’s research group. To minimize potential bias, independent clinical studies, systematic reviews, practice guidelines, and methodological reports were incorporated wherever available. Accordingly, the findings presented here should be interpreted as part of a growing body of evidence that complements established knowledge rather than replacing it. Further independent studies will be important to confirm the generalizability and clinical relevance of imaging-derived measures of nutrient persistence.

A key challenge for the field is to move beyond proof-of-concept visualization and develop imaging metrics that can be linked to meaningful outcomes. Preclinical imaging provides an opportunity to quantify nutrient persistence over time and to examine how formulation design, device characteristics, and GI retention affect nutrition delivery and feeding tolerance. Imaging-based approaches may also help improve standardization and reproducibility as well as facilitate cross-study comparisons in preclinical nutrition research. Establishing standardized imaging protocols and outcome-linked metrics will be an important step toward broader adoption and clinical translation. Future work should focus on establishing consistent imaging protocols and determining whether residue and retention metrics are associated with outcomes such as catheter dysfunction, microbial proliferation, feeding intolerance, and nutrient delivery efficiency. Ultimately, validation across independent laboratories, experimental models, and clinical settings will be essential for translating nutrient persistence from an imaging observation into a useful biomarker.

## 6. Future Directions

Future research should focus on advancing both the methodological robustness and translational applicability of imaging-based approaches to nutrient persistence. Emerging from the current evidence are several priorities.

First, the development of improved imaging probes is essential. While ICG has proven practical, next-generation fluorophores with improved stability, specificity, and optical properties are needed to support long-term monitoring and broader applications in fluorescence-based imaging. Such advances may enable more precise characterization of nutrient behavior across different clinical contexts [36,37,38,39,40,41].

Second, integration of imaging with device engineering and formulation science represents a critical opportunity. Systematic evaluation of catheter geometry, flow dynamics, and formulation rheology could further inform the design of PN and EN systems with improved nutrient clearance [16,17].

Third, establishing standardized protocols and reporting frameworks may improve reproducibility and comparability across studies. Consensus on acquisition parameters, signal quantification methods, and data reporting standards would enable cross-study validation and facilitate the accumulation of robust evidence suitable for meta-analysis [40,41,43].

Fourth, future preclinical imaging studies should be designed with explicit translational endpoints. Imaging-derived residue and retention metrics should be linked to clinically relevant outcomes, including catheter performance, microbial risk, feeding tolerance, and efficiency of nutrient delivery. Such studies should incorporate clinically relevant models and standardized imaging protocols so that imaging-derived metrics can be compared across laboratories and connected to patient-oriented outcomes. This outcome-linked approach would help shift preclinical imaging from proof-of-concept visualization toward a translational tool for improving clinical nutrition practice [40,41,43].

Collectively, these directions could help move imaging-based approaches from experimental methodologies toward standardized, clinically useful tools for optimizing nutrition delivery across PN, EN, and GI systems.

## 7. Conclusions

This review highlights nutrient persistence as a central and previously underrecognized determinant of performance across parenteral, enteral, and GI nutrition systems. By synthesizing evidence from device-level, system-level, and physiological studies, it demonstrates that residue formation and prolonged retention arise from shared principles related to transport dynamics, formulation properties, and physicochemical transformations.

Fluorescence-based imaging enables spatially resolved visualization and quantification of these processes, transforming nutrient persistence from a largely inferred phenomenon into a measurable biological parameter. These approaches clarify how transport dynamics and device geometry affect residual accumulation in parental nutrition systems, how formulation viscosity and thickener chemistry shape residue formation and microbial co-localization in EN tubing, and how physicochemical transformations within the GI tract determine transit behavior and prolonged retention.

Together, these insights support a shift toward evidence-based optimization of nutrition delivery, in which device design, formulation characteristics, and physiological responses are considered within a unified framework. As imaging technologies continue to evolve, they may contribute to improving safety, enhancing feeding tolerance, and informing the development of evidence-based nutritional strategies.

## Figures and Tables

**Table 1:** Comparison of imaging and non-imaging approaches for assessing gastrointestinal nutrient retention and transit.

Method	Principle	Strengths	Limitations	Translational Relevance	3Rs Relevance
MRI [30,31]	Anatomical and functional imaging	High spatial resolution; no ionizing radiation; suitable for repeated assessment	High cost; limited throughput; specialized equipment required	High clinical relevance for gastric emptying and motility assessment	Supports longitudinal assessment and may reduce repeated animal use
Scintigraphy [32]	Radiolabeled meal tracking	Established clinical reference method; quantitative assessment of gastric emptying	Radiation exposure; specialized facilities required; limited repeatability	Strong clinical comparability	Limited repeated measurements because of radiation exposure
Dye/bead marker assay [33,34]	Endpoint-based transit assessment	Simple, inexpensive, and widely accessible	Low spatial resolution; often endpoint-based; limited temporal information	Limited direct clinical translation	May require separate animal cohorts or terminal sampling
13C breath test [35]	Metabolic surrogate of gastric emptying	Non-invasive; clinically established	Indirect measurement; affected by absorption, metabolism, and analytical conditions	High clinical applicability for gastric emptying assessment	Supports repeated measurements, but requires specialized analytical systems
Fluorescence imaging	Optical tracking of labeled or intrinsically fluorescent nutrients	High sensitivity; spatially resolved; suitable for longitudinal assessment in experimental models	Limited tissue penetration; probe dependence; signal attenuation; standardization challenges	Useful translational bridge between benchtop, preclinical, and clinical research	Supports Reduction and Refinement; may contribute to partial Replacement through benchtop optimization

Footnote: Fluorescence imaging should be regarded as complementary rather than universally superior to established methodologies. The optimal method depends on the research question, target tissue depth, required temporal resolution, quantitative needs, and clinical or experimental setting.

## Data Availability

No new data were created or analyzed in this study. Data sharing is not applicable to this article.
